# Review of the Role of Nanotechnology in Overcoming the Challenges Faced in Oral Cancer Diagnosis and Treatment

**DOI:** 10.3390/molecules28145395

**Published:** 2023-07-14

**Authors:** Vidhya Rekha Umapathy, Prabhu Manickam Natarajan, Bhuminathan Swamikannu

**Affiliations:** 1 Department of Public Health Dentistry, Thai Moogambigai Dental College and Hospital, Dr. M.G.R. Educational and Research Institute, Chennai 600107, Tamil Nadu, India; 2 Department of Clinical Sciences, Centre of Medical and Bio-Allied Health Sciences and Research, Ajman University, Ajman P.O. Box 346, United Arab Emirates; 3 Department of Prosthodontics, Sree Balaji Dental College and Hospital, BIHER University, Pallikaranai, Chennai 600100, Tamil Nadu, India; bhumi.sbdch@gmail.com

**Keywords:** oral cancer, diagnosis, treatment, nanotechnology, nanoparticles

## Abstract

Throughout the world, oral cancer is a common and aggressive malignancy with a high risk of morbidity, mortality, and recurrence. The importance of early detection in cancer prevention and disease treatment cannot be overstated. Conventional therapeutic strategies have minor difficulties but considerable side effects and unfavourable consequences in clinical applications. Hence, there is a requirement for effective ways for early detection and treatment of oral cancer. At present, numerous forms of nanoparticles have piqued researchers’ interest as a potentially useful tool for diagnostic probes and medicinal devices. Because of their inherent physicochemical properties and customizable surface modification, they are able to circumvent some of restrictions and accomplish the intended diagnostic and therapeutic impact. Nanotechnology is a unique field that has revolutionised the industry and is paving the way for new treatments for oral cancer. It can help with a better diagnosis with less harmful substances and is setting current guidelines for treatment. The use of nanotechnology in cancer diagnosis, therapy, and care improves clinical practise dramatically. The different types of nanoparticles that have been developed for the diagnosis and therapy of oral cancers will be covered in this study. The difficulties and potential uses of nanoparticles in the treatment and diagnosis of oral cancer are then highlighted. In order to emphasise existing difficulties and potential remedies for oral cancer, a prospective view of the future is also provided.

## 1. Introduction

Cancer is a serious global public health issue that has imposed a significant impact on society. Oral cancer (OC) is the sixth most predominant cancer in the world, and has a 50% 5-year survival rate [1]. This disease has a propensity for rapid spread and frequently exhibits metastases and invasion of nearby tissue. Aggressive OC primarily affects oral epithelial cells, has a chance of metastasizing, and can even be fatal [2]. OC may result in psychological distress, dysphagia, paraesthesia, altered facial appearance, chronic pain, and other symptoms [3]. Oral squamous cell carcinomas (OSCC), which make up more than 90% of all oral malignancies, are the most common kind of malignancy [4]. The tongue is said to be the most prevalent subsite of OC, with a bad prognosis, and they may also infiltrate the buccal, floor of the mouth, alveolar, and hard palate [5,6]. There are a number of significant risk factors for OC, with smoking serving as the primary cause of cancer death [7,8], along with alcohol intake and human papillomavirus (HPV) infection. Additionally, regular use of areca nuts is another risk factor for OC, particularly in the Indian subcontinent [9]. OC development is a complicated, multi-step process [6]. Oral leukoplakia, oral erythroplakia, oral lichen planus, oral submucous fibrosis, actinic keratosis, and discoid lupus erythematosus are the most common oral potentially malignant disorders (OPMD) with the potential to progress to malignancy [9,10].

Long and expensive diagnostic procedures frequently fail to effectively distinguish between normal and tumour tissue, which may cause a delay in the start of treatment. The standard diagnostic techniques used to identify OPMD and OC are scalpel biopsy and histological exams [11,12]. However, the biopsy procedure is frequently intrusive; patients may feel uneasy and uncomfortable [13]. The choice of resection margins is mostly determined via histological evaluations, and the accuracy of the pathologists’ assessments and the quality of the samples may have an impact on the outcomes [14,15]. Additionally, there are several restrictions and side effects associated with conventional treatments for OC, such as surgery, radiotherapy, and chemotherapy [16]. Therefore, it is essential to improve diagnosis accuracy and lessen therapeutic adverse effects. 

Nanotechnologies have been used in several industries over the past few decades, particularly in the field of medicine in order to monitor the spread of disease and to diagnose malignancy [17,18,19,20,21]. Nanomedicine, a part of nanotechnology which increased the likelihood of precise cancer therapy, has received the most attention [22]. A variety of cancers, including cervical cancer, lung cancer, breast cancer, stomach cancer, nasopharyngeal cancer, and OC, have been detected and diagnosed using nanotechnology [23,24,25,26,27,28]. Biomaterials with special (bio)physicochemical properties have been shown to have the ability to facilitate cell activity and even treat diseases, restore biological functions, or regenerate tissues [29,30,31,32,33,34,35,36,37,38]. Particularly, the synthesis of nanomaterials and their broad use in biomedical diagnostic and therapeutic procedures have drawn considerable interest [39,40,41,42,43,44]. The majority of NPs created today are passively targeted and based on the enhanced permeability and retention (EPR) effect for the detection and treatment of cancer. More precise and effective active targeting techniques are required due to the unpredictability of the EPR effect in the tumour microenvironment. Additionally, there is hope for the detection and treatment of cancer thanks to a developing nanotechnology-based methodology that uses biosensors [45,46] and gene therapy [47]. Due to their ultrasmall size, high reactivity, and tuneable functional modification [48,49], both organic and inorganic NPs have been used extensively [50]. This study outlines recent developments in the formation of various NPs and their use in the detection and treatment of OC. This review is anticipated to assist researchers in better understanding the impact of NPs on the detection and treatment of OC and may hasten technological advances in this area.

## 2. Oral Carcinogenesis

Multistep carcinogenesis, the process through which a normal cell transforms into a cancer cell, is well known to be the result of an accumulation of genetic changes. The onset and development of OC entail a variety of complex processes. The development of OC follows a similar multistep process to that of other malignancies, including the accretion of several genetic and epigenetic modifications that are influenced by genetic predisposition and risk factors. Age, gender, alcohol and cigarette usage, chewing tobacco or betel quid, exposure to radiation, HPV infection, inflammation, etc. are risk factors for OC [51,52,53]. The molecular regulation of these numerous pathways is becoming better understood as a result of technological advancements. These, in turn, might aid in more precise diagnosis, prognosis evaluation, as well as more inventive approaches to therapy and prevention.

## 3. Challenges in OC Diagnosis 

Despite the oral cavity being a potentially accessible area for assessment, up to 50% of OCs are not discovered until the disease is well established [54]. One of the most effective approaches to lower the high death rate from OC is early identification. Early detection increases the likelihood of OC survival to more than 90% after five years, compared to only 30% for patients who present at a late stage [55]. Early detection can reduce the disease’s morbidity, which is linked to a serious loss of function, deformity, depression, and a poor quality of life, as well as the cost of therapy. Early detection may be helped via screening, which could also benefit patient outcomes. Unfortunately, the majority of patients (almost 60%) have an advanced stage of cancer. The main reason that the OC survival rate has not improved significantly has been linked to a delay in diagnosis that lengthens the period before therapy can begin. 

## 4. Challenges in OC Treatment 

Surgery, radiation, chemotherapy, antibody blocking therapy, or a combination of therapies are the mainstays of treatment for OC. The location, size, stage, and type of the cancer, as well as its progression and the patient’s overall health, all affect the suggested treatment modality or combination of treatment modalities [56]. There are considerable limits to the OC treatments that are now available. Resection in OC surgery is constrained by a number of nearby crucial structures, including the carotid artery, the eye, and the brain. Adjuvant therapies are required in some situations to cure the remaining disease, such as when the residual tumour is near essential tissues or has progressed past the surgical margin. In terms of radiation therapy, it frequently fails to treat advanced tumours. Furthermore, the amount of radiation that can be used for a whole course of treatment is constrained by toxicity. In OC, chemotherapy is only used in conjunction with radiation therapy to provide supportive care. Chemotherapy given concurrently with radiation therapy is more effective than chemotherapy given before a session of radiation therapy. If the cancer is progressed (stage III or IV), chemotherapy is added to the radiation treatment programme [56,57]. Anticancer medications, such as cisplatin, cetuximab, fluorouracil, paclitaxel, docetaxel (DTX), and methotrexate, are being used in OC chemotherapy treatments [57,58,59,60,61,62,63,64,65,66,67]. This is merely a list of some chemotherapy drugs. Additionally, doctors may decide to employ other relevant medications. 

Intravenous delivery is the most direct method. It can achieve immediate and full bioavailability and overcome the gastrointestinal tract’s varied absorption patterns. High drug concentrations are supplied to healthy tissues with this mode of administration, which is one of the potential risks. This could exacerbate unpleasant reactions and promote harm to normal, healthy tissues [68]. In people with OC, complications are frequent. Complications are new medical issues that develop during or after a condition, procedure, or treatment that make recovery more challenging. The side effects of the condition or its treatment may create the complications, or they may have other underlying causes. Because of a few factors, OC patients in particular have a greater risk of oral problems. Chemotherapy and radiation therapy have the effect of reducing or halting the growth of rapidly proliferating cells, including cancer cells. Normal cells in the lining of the mouth are also quickly proliferating cells inside the human oral cavity. Anti-OC therapies can thus also prevent the growth of these healthy cells. This may hinder the ability of oral tissue to regenerate new cells and repair itself. Radiation therapy, on the other hand, is more likely to directly harm and degrade bone, salivary glands, and oral tissues. Furthermore, the healthy balance of bacteria in the mouth might be upset by chemotherapy and radiation therapy. There are many distinct types of both helpful and dangerous bacteria in the oral cavity.

## 5. Nanotechnology

Because of the nature of the materials they are made of, nanoparticles might possess characteristics including self-assembly, stability, specificity, drug encapsulation, and biocompatibility [69]. The optical, magnetic, catalytic, thermodynamic, and electrochemical capabilities of NPs are just a few examples of their unique size-dependent physical and chemical characteristics. The National Institutes of Health (Bethesda, MD, USA) has termed this field of study “Nanomedicine” because of the significant potential for therapeutic usage of these particles. Thus, the early diagnosis of tumours and the treatment of OC are both made possible by nanotechnology. Quantum dots (QDs), carbon nanotubes, paramagnetic NPs, liposomes, and gold NPs are examples of well-studied NPs [70]. The advancement of biomaterials and improved surfaces for medical devices, in vitro and in vivo approaches, biofiltration systems, robotic assembly, and many other fascinating developments are all made possible by nanotechnology, which is revolutionising biomedical engineering. Numerous advancements and breakthroughs in oral disease prevention, diagnosis, and therapy are made possible by nanotechnology [71].

## 6. Nanoparticles 

Ultradispersed solid supramolecular structures having a size between 10 and 1000 micrometres are known as nanoparticles (NPs) [72,73]. NPs are well suited in size to the biologic substances and structures present inside living cells on the size scale of 1–200 nm. As a result, NPs seem to be the right size range for molecular imaging and manipulation. A few nanometres in diameter, NPs are sphere-shaped biocompatible substances comprised of inert silica, metal, or crystals. The development of NPs requires the coacervation of hydrophilic polymers, the polymerization of monomers, the dispersion of preformed polymers, and ionic gelation. Other techniques for producing NPs have been reported, including supercritical fluid technology and particle reproduction in nonwelting templates [74,75,76,77].

The controllability of an NP’s surface is a benefit. This enables conjugation to different ligands for molecular interactions. These NPs, which are between 100 and 10,000 times smaller than human cells, have the power to interact in previously unheard-of ways with proteins both on and inside of cells. This has the potential to completely alter how cancer is detected and treated. In comparison to conventional therapies, NPs have the capacity to boost anticancer effects while concurrently reducing systemic toxicity. This is because they are more selective and specific toward the targets. NPs can improve the stability of pharmaceuticals and provide tailored delivery management. This will enhance drug extravasation into the tumour system and, in turn, allow for a consistent and uniform concentration at the location of a lesion, reducing adverse effects [78,79,80]. Additionally, NPs have the ability to circumvent medication resistance, resulting in increased intracellular drug accumulation.

### 6.1. Types of NPs 

An appropriate stabiliser produces and synthesises nanosuspensions, which are colloidal dispersions of drug particles with a diameter of less than one nanometre. The size is between 30 and 100 nm. There are two different kinds of NPs: nanospheres and nanocapsules. Medication is dissolved and disseminated in polymer matrices called nanospheres. The oil-filled centre of a nanocapsule’s polymer walls is where the medicine is dispersed. They benefit from increased effectiveness, lower harmful levels, better distribution, and more compliance [81].

NPs work with innate immune systems to trigger an anticancer immune response. Through identifying and removing damaged cells and proteins and providing an immediate line of defence against external invaders, innate immunity aids in maintaining the body’s integrity. Numerous in vitro and in vivo experimental animals have been used to study the interaction of NPs with monocytes and macrophages in a variety of biological types. This is dependent on how the substance enters the body and whether microenvironmental proteins “cover” the reactive NP surface as a result. 

### 6.2. Properties of NPs 

NPs have three layers: a core, a surface layer, and a shell layer. Surfactants, polymers, and metal ions make up the surface layer. There are lipid, polymeric, ceramic, nonmetal, semiconductor, and metallic NPs. The available sizes and material type affect the properties [82]. NPs that are smaller than 50 nm are considered to be super hard. Also different are the malleability and ductility. Some magnetic materials exhibit super paramagnetism, whereas others exhibit surface plasmon resonance and quantum confinement Q-particles. Due to their large surface area to volume ratio, some exhibit solar radiation in photovoltaic cells and improved diffusion at high temperatures. Another characteristic of NPs is that they start localised surface plasmon resonances at wavelengths that are close to infrared, which improves image contrast and resolution [83].

The function of nanoparticles in composite material structures and their advantages for bodily health are valued at the moment. Using a Mg co-doped ZnO hydrogel that was created using the sol–gel method, Sabbagh et al. (2020) investigated the impact of doping on the structural and morphological features of hydrogels [84]. Then, as a control, a hydrogel with nanoparticles and a hydrogel without any nanoparticles were created. The hydrogels included a lot of catechin, and the associated characterization was developed using the novel matrix structure. Zeta potential, XRD, and the nanoparticles’ characteristics were investigated. The hydrogels with nanoparticles had a more compressed structure, according to FESEM data, than the hydrogels without one. The hydrogels containing nanoparticles had a higher breakdown temperature on the TGA. They discovered that the synthesised nanoparticles’ function in the hydrogel’s structure is crucial.

Using the sol–gel method and calcination temperatures of 650 °C, Mahmoudi Khatir et al. (2016) produced Zn_1−x_Fe_x_O NPs with various Fe concentrations and structurally characterised them to ascertain their antibacterial activities [85]. The presence of wurtzite structure in Zn_1−x_Fe_x_O NPs without any pyrochlore phase was shown in the XRD pattern. The size–strain plot approach and the Scherrer formula are used to analyse the considerable widening in the XRD peak. With an increase in Fe concentrations, it has been seen that the diameters of the nanocrystallites (between 30 and 45 nm) shrink. It was discovered that adding Fe atoms in amounts less than 5% did not significantly increase the antibacterial activity of ZnO NPs; instead, it only gradually increased by 5%. The enhancement in antibacterial activity of ZnO NPs due to low-percentage Fe doping cannot be inferred to be beneficial.

## 7. Cancer Nanotechnology 

The Human Genome Project has produced a wealth of knowledge about cancer genomics and proteomics, according to the US National Cancer Institute, offering vital information about how cancer progresses. As a result, there are now more chances to address cancer’s molecular information. Despite the fact that a number of medications have been introduced with the goal of curing cancer, the majority of them have turned out to be either too toxic or just ineffective at increasing lifespan as predicted. Most of these medications have downsides in the form of side effects. These medications affect both normal and tumour cells because of their nonspecificity. However, technical advancements are still needed to translate promising molecular findings into benefits for cancer patients. Here, nanotechnology can be extremely important since it will give those working on new diagnostics, treatments, and preventives the technological capacity and resources they need to keep up with the present increase in information. The major challenge in any cancer therapy is to deliver the right amount of drug to the tumour locations, where it can kill cancer cells while causing the least amount of harm to healthy cells. With this as the goal, it is essential to develop single drug that has the potential to significantly impact cancer prevention, detection, and therapy. The theory underlying targeted delivery is that chemotherapy medications can be directed to cancer cells through taking advantage of the same characteristics of cancer cells that made their identification and targeting possible. 

NPs are important for treating cancer in two different ways. The first function of NPs is as drug carriers. Second, various light wavelengths can be absorbed by NPs. NPs heat up when exposed to the right wavelengths, killing cancer cells only while avoiding heating up the rest of the body. NPs may enter the smallest capillary veins because of their extremely small volume, which allows them to avoid being quickly cleared by phagocytes, considerably extending their time in the bloodstream. Their ability to reach target organs by means of passing through cell and tissue gaps is another quality. They can exhibit controlled release qualities because of their materials’ biodegradability, pH ion sensitivity, and temperature sensitivity. They can be tuned for certain surface qualities. It is possible to attach water-insoluble anticancer medicines to injectable NP solutions without employing hazardous organic solvents. Both passive and aggressive tumour targeting can be accomplished using NPs. They are good podiums for cancer detection, imaging, and treatment because of their characteristics [1,86]. 

Nanorobots can be used in chemotherapy to accurately administer the right quantity of chemotherapeutic chemicals to target cells, fighting cancer. Nanorobots are able to identify sick cells and can locate and eliminate them wherever they are. Given that normal cells would be spared, this technique would be more effective and have less adverse effects [87,88]. Cancer nanotechnology is a rapidly developing area of interdisciplinary study that promises to significantly increase cancer screening, diagnosis, and treatment. The concept of manipulating drugs/chemicals at the nanoscale to create more potent cancer treatments is anticipated to offer a convincing solution for the selective destruction of cancer cells while causing minimal harm to healthy cells.

Liposomes, polymer microspheres, protein conjugates, and polymer conjugates are only a few of the nanoformulations for cancer therapies now being used in clinical settings. Novel nanomaterials are also being studied for improved medication effectiveness and targeting [89]. Since it may greatly reduce the toxicity associated with non-specific activity, targeted delivery is the ultimate in cancer therapy, as was previously stated. Clinical studies are now being conducted on a number of novel innovations that entail targeting moieties. Targeted drug release has the potential to reduce overall toxicity and the minimal effective dose even further, enhancing effectiveness and patient quality of life [90]. Therapeutics can be created to obtain the best efficacy and the least amount of toxicity when technology develops to use specialised delivery. While certain targeted medicines may show tumour selectivity, their clinical efficacy may be constrained by their PK/PD or biodistribution characteristics. The efficacy and specificity of the anti-cancer drug tumour necrosis factor-related apoptosis-inducing ligand (TRAIL), which targets malignant cells while sparing healthy ones, make it the best treatment for cancer [91]. The often-targeted TRAIL has a brief half-life and a quick renal clearance, which makes it difficult to go through preclinical [92]. Any therapeutic’s PK/PD characteristics might be improved through nanoformulation, which would allow for drug repurposing [93].

Newer nanomaterials might improve cancer immune therapy further. For instance, Gram-negative bacteria release outer membrane vesicles (OMVs), which are sized 30–250 nm and act as a mediator of bacterial communication and homeostasis [94]. They have ideal qualities for vaccine distribution, including small size and simplicity of scaling up manufacture, and they have inherent immunostimulatory capabilities [95]. It has recently been demonstrated that tumour antigens can appear as ClyA fusion proteins on OMV surfaces and trigger T-cell-mediated, targeted anti-tumour response [96]. Additionally, a protein tag can spontaneously link to the protein catcher utilising the protein “Plug-and-Display” technique through forming an isopeptide bond. 

For the simultaneous administration of many chemotherapeutic drugs, nanomaterials have proven to be highly helpful. The co-delivery of drugs inside a single carrier can normalise distribution and delivery since drugs have a variety of biochemical characteristics that might be significantly different from their synergistic complement [97]. The immune checkpoint is now disrupted in the clinic with anti-PD-1/PD-L1 antibodies, which reverse T cell dysfunction and exhaustion and effectively treat cancer [98]. Recently, it was shown that a liposomal formulation of the histone demethylase inhibitors 5-carboxy-8-hydroxyquinoline (IOX1) and DOX significantly lowers tumour immunosuppressive factors and enhances T cell infiltration and activation [99]. Studies conducted in vivo revealed that different mice tumours (including those that metastasized to the subcutaneous, orthotopic, and pulmonary tissues) grew less rapidly and that the treatment provided a long-lasting immune memory capacity to protect against tumour recurrence. The results of the study demonstrated that IOX1 suppresses P-glycoproteins (P-gp) in cancer cells via the JMJD1A/-catenin/P-gp pathway and synergistically increases DOX-induced immune-stimulatory immunogenic cell death. According to the intended outcome, drug release may be optimised using nanoformulation through adjusting release kinetics for dual-drug loading [100]. Drugs may be released in a variety of ways, and because of this, the release rate can be quite particular to increases in stimuli responsiveness [101]. Drug-free carriers revealed modest cytotoxicity towards MCF-7 cells. Additionally, the extracellular release of PTX increased the interstitial space, which, through a tumour priming effect, enabled deeper penetration of the NPs into the tumour mass. This led to better in vivo circulation time, tumour-targeted administration, and overall therapeutic effectiveness for FPL-PMSN-PTX/ATO.

## 8. NPs for OC Therapy 

Due to their adaptable chemical and physical properties, NPs are becoming more and more common in targeted drug delivery systems with improved bioactivity and successful therapy, lowering their systemic toxicity for the treatment of OC. The chemotherapeutic medicines can be loaded, stabilised, and delivered using these carriers, which mostly consist of polymeric and inorganic NPs, in a variety of loading contents and release profiles [102]. 

### Liposomes for OC Diagnosis and Therapy

**Figure 1:**
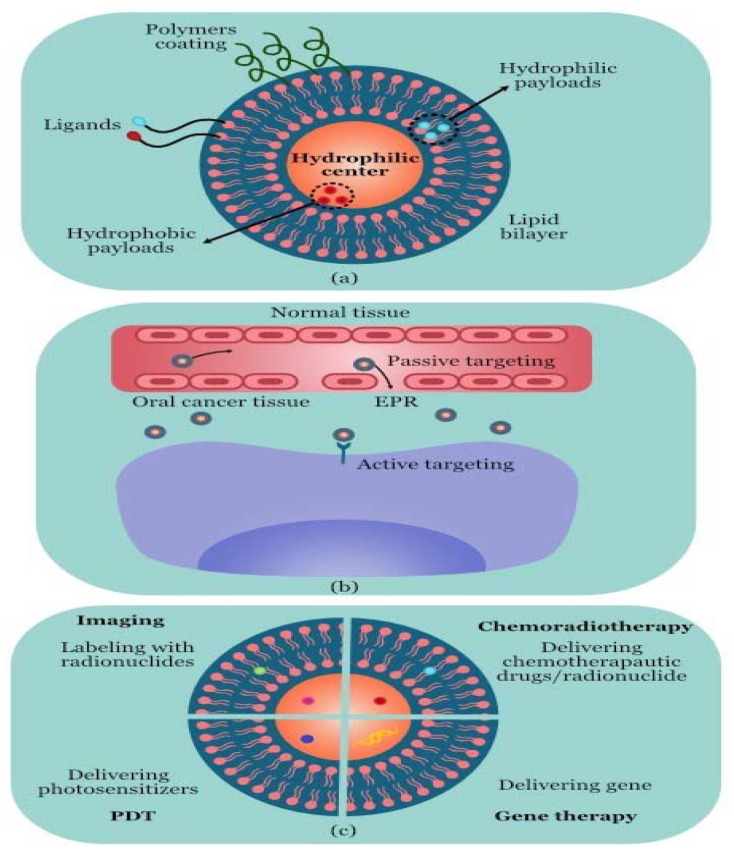
Liposome-based diagnosis and treatment of oral cancer. (**a**) Structure of liposome; (**b**) liposomes invading cancer tissue; (**c**) different treatment processes.

Liposomes are microscopic unilamellar or multilamellar particles made of membrane-like lipid layers that are frequently formed of phospholipids and cholesterol and surround aqueous compartments (Figure 1) [103,104]. For the systemic administration of numerous medications to reduce drug toxicity and increase drug accumulation at target areas, liposomes are the most used drug delivery mechanism [105]. In order to increase therapeutic effectiveness and reduce damage to normal cells, liposomes have been extensively researched for the transport of chemotherapeutic medicines [106]. Liposomes are closed, spherical particles with a hydrophilic centre comprised of amphiphilic phospholipid bilayers. Payloads that are either hydrophilic or hydrophobic can be enclosed in the lipid bilayer or the hydrophilic centre, respectively. Using the EPR effect as a guide, passive targeting was used to introduce liposomes into tumour tissue. Additionally, ligands are added to the liposome surface for specific targeting. For instance, high-affinity folate-bound liposomes can preferentially target the tumours’ folate receptors [107]. The stability properties and encapsulation effectiveness of various liposomal formulations varied significantly, which can be used to meet specific diagnostic and therapeutic demands [108]. 

In the investigation of cancer diagnosis, liposomes have frequently been utilised. For instance, the combination of a radionuclide and an anchor molecule that is located inside the hydrophilic core or enclosed in the phospholipid bilayer has frequently been used to achieve labelling with radionuclides like 64Cu. Compared to 18F-FDG, 64Cu liposomes may be able to detect early stage tumours, according to research by Mahakian and group [109]. 

Liposomes are commonly employed in delivery systems for a range of anticancer therapies to increase the efficiency of anticancer drugs and reduce adverse effects. The surfaces of drug-loaded liposomes have been coated with a variety of biocompatible polymers, including polyethylene glycol (PEG), due to the ease with which the reticulo-endothelial system (RES) may remove them. PEG is a man-made hydrophilic polymer that can be crosslinked with the liposome surface to prolong the half-life in the bloodstream and prevent RES removal. Numerous studies have been conducted on formulations created through adding antitumour medications, including doxorubicin, to liposomes. PEGylated liposomal doxorubicin (PLD) has a stronger apoptotic effect on CAL-27 cells than free doxorubicin [110]. Although PLD has a strong safety record, certain patients’ long-term usage of the drug has been linked to the emergence of OSCC or precancer lesions [111,112]. Curcumin, paclitaxel, carboplatin, and cisplatin are other medications included in liposomes and are similarly more effective at inducing the apoptosis of cancer cells [113]. It was discovered that taking two or more anticancer medications with supplements increased the effectiveness of chemotherapy. In the case of OC, the coencapsulated liposomal formulation of doxorubicin (Dox) and resveratrol (Res) has been investigated, and in vitro testing of the medication combination revealed improved efficacy in the treatment of OC [114]. Meanwhile, liposome-based gene therapy formulations have considerable promise for the treatment of OC. A straightforward method of introducing therapeutic genes into target cells, liposomes represent a promising substitute for viral vectors [115,116]. The use of liposomes as radionuclide carriers for tumour radiation has also been investigated. After convection-enhanced administration, 186Re loaded into liposomes can successfully cure OC with few negative effects [117]. 

## 9. Dendrimers for OC Diagnosis and Therapy 

**Figure 2:**
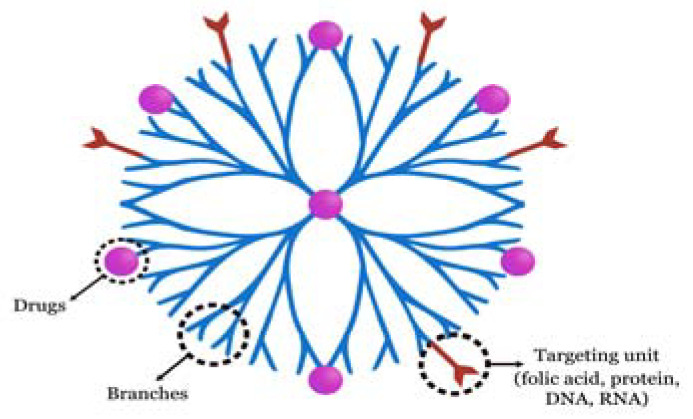
Structure of dendrimers for OC diagnosis and treatment.

A central core, repeated branches, and terminal functional groups are the three main parts of the 3D, multibranched, and tree-like dendrimers (Figure 2) [118]. Dendrimers are typically synthesised primarily using the divergent approach and the convergent method. Dendrimers have up till now been extensively used in a variety of fields, including electrochemistry, medication delivery, and gene transfection.

Dendrimers are desirable devices for the detection of oral malignancies. Wei et al. [119] created DNA-dendrimer and polypyrrole (DDPpy) sensors with improved selectivity and stronger bioaffinity to detect OC biomarkers such interleukin-8 RNA, interleukin-8 protein, and interleukin-1 protein. Drugs are either covalently conjugated to the terminal functional groups or enclosed in the inner cavity. As a result, dendrimers are a popular choice for medication delivery. When used in xenograft tumour growth models, coencapsulating methotrexate (an anticancer treatment) and folic acid (a targeting agent) improved tumour control compared to free drugs [120]. The transfection of gene agents could be one step farther. In OSCC cells and xenograft mouse models, polyamidoamine (PAMAM) dendrimer-mediated shRNAs can silence human telomerase reverse transcriptase (hTERT), indicating the effectiveness of this system in cell apoptosis and tumour growth inhibition [121]. 

## 10. Magnetic NPs for OC Diagnosis and Therapy

**Figure 3:**
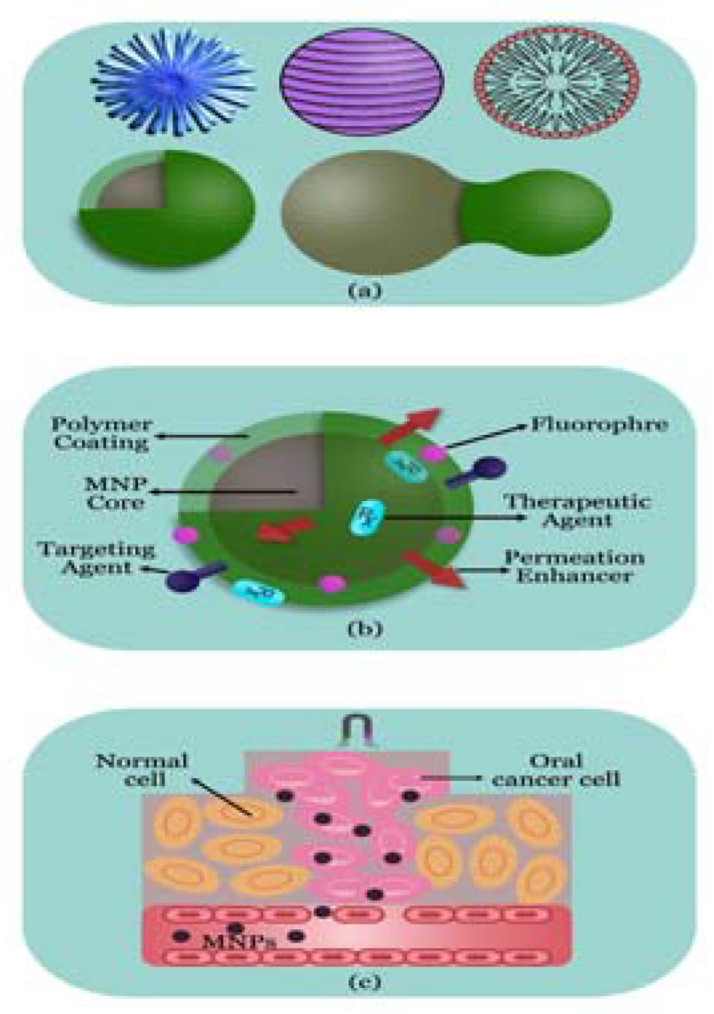
MNP-based OC diagnosis and treatment. (**a**) Structure of MNP; (**b**) multifunctionality of MNP with different ligands; (**c**) therapeutic effect.

MNPs come in a variety of sizes and alterations, and they all have excellent magnetic characteristics, good stability, and are biocompatible and biodegradable (Figure 3). There are numerous uses for magnetic resonance imaging (MRI), drug delivery methods, and hyperthermic cancer treatment, including for OC. Co-precipitation, thermal decomposition, microemulsion, hydrothermal, combustion, and polyol syntheses are only a few of the numerous techniques that have been established to date to create MNPs [122,123]. However, the uses of MNPs are still restricted because of the RES’s capacity for absorption and tendency to aggregate. Polymeric coatings act as a barrier to stop RES uptake and stop the aggregation of NPs [124]. 

Today’s clinical medicine uses MRI as the most beneficial noninvasive imaging modalities. As MRI contrast agents, MNPs are actively being researched. They can aid in proton relaxation refinement and eventually grow into valuable probes for contrast in both medical and biological diagnostic applications [125]. MNPs can also be administered into the tumour site with precision, avoiding adjacent organs [126]. MNPs have been used to segregate the pharmaceuticals selectively to the cancer site under the control of an external magnetic field, making them as the most promising targeted drug delivery systems. MNP system was made using the solvothermal process, and the surface of MNP was changed using polyacrylic acid (PAA) to improve the loading quantities of bleomycin (BLM) [127]. Under a magnetic field, BLM-MNPs continuously accumulated in tumour tissue and, through slowly and locally releasing BLM, prevented the growth of the tumour. 

Apoptosis is induced by the inhibition of B-cell lymphoma-2 (BCL2) and baculoviral IAP repeat-containing 5 (BIRC5). Based on the Fe_3_O_4_ NPs, siRNA-targeting BCL2 and BIRC5 delivery system [128]. The polyethyleneimine (PEI) coating on MNP supplied a positive charge that is required for siRNA capture and functions as gene silencing following cellular absorption. Miao et al. [129] employed PEI-modified Fe_3_O_4_ NPs driven by the hTERT tumour-specific promoter to target to the human-TRAIL gene and cause apoptosis. Comparatively speaking, cancer cells exhibit higher hyperthermia sensitivity than normal cells. PTT has the ability to stop tumour cells from proliferating while also causing their deterioration and necrosis. Although PTT using gold NPs can destroy OC cells, PPT is typically an effective treatment for superficial tumours. Under an alternating magnetic field, magnetic fluid hyperthermia can produce heat via physical rotation and rotation of magnetic vectors, resulting in cell apoptosis and irreversible cellular damage. Legge et al. [130] created integrin-v6-targeting magnetic iron oxide NPs that are biocompatible, coated in silica, and coupled with antibodies to treat OSCC. 

## 11. Quantum Dots for OC Diagnosis and Therapy 

Quantum dots (QDs) are semiconductor nanocrystals that typically have a diameter between 1 and 10 nm and are made up of elements from groups II–IV, IV–VI, or III–V [131]. QDs have strong fluorescence intensities, narrow emission spectra, broad absorption spectra, and broad excitation spectra from ultraviolet to near-infrared (or between 450 and 850 nm). In cancer therapy, QDs can be used as probes or drug delivery vehicles, but they can also produce reactive oxygen intermediates or species (ROIs/ROS) or heat when exposed to radiation to kill cancer cells [132].

Nanometre-sized semiconductor crystals known as QDs emit light due to quantum conflation effects [133,134]. QDs have a number of benefits that could help them overcome the drawbacks of traditional fluorescent dyes, including size-tuneable emission, broad excitation spectra, high luminescence, and exceptional photobleaching stability [134,135,136]. Additionally, altering the size and makeup of QDs enables the production of a broad spectrum, from ultraviolet to near-infrared [137,138]. The majority of the compounds that may target cancer cells were attached to the QDs that were utilised for in vivo imaging [139]. Near-infrared QDs with an emission wavelength range of 700–900 nm have recently been found to exhibit significant tissue penetration and to be safe in vivo [139,140]. Meanwhile, tissue autofluorescence can be avoided by means of using QDs with emission wavelengths between 400 and 600 nm, making them appropriate for bioimaging [141,142]. 

Compared to conventional organic fluorescent materials, QDs offer better optical characteristics because of their distinct quantum size and surface effects. Zhao et al. tagged Tca8113 cells using FITC and QDs (goat anti-rabbit Qd655 nm-igG), noting that QDs are better suited for long-term dynamic observation of cellular physiological changes than FITC due to their superior fluorescence intensity and photostability [143]. The accurate optical qualities of QDs have recently been extensively developed for the detection of cancer cells. In an OSCC animal model, Yang and Chen created EGFR-antibody-conjugated QD800 (QDs with a maximal emission wavelength of 800 nm) for the targeting and in vivo imaging of the human buccal squamous cell carcinoma cell line (BcaCD885). QD800 has strong tissue penetration and is suitable for visible fluorescence imaging [144]. Through conjugating QDs with molecular biomarkers, multifunctional NP probes have been created. These QD probes can be used to find out which molecular biomarkers are expressed in OC cells. In addition, it is difficult to identify targets with high sensitivity when cells contain such tiny amounts of protein and nucleic acids, necessitating the use of QDs with high binding specificity. In order to investigate the relationship between OSCC and the human papillomavirus (HPV), QD in situ hybridization (QDISH) was used and the results showed that QDISH had higher sensitivity than ISH [145]. A noteworthy advancement in recent years that may help with the detection of some biomarkers is the biosensor based on QDs. 

The recently created fluorescent NPs known as carbon QDs (CQDs) are constructed of carbon, which guarantees exceptional biocompatibility and persistent fluorescence. A N-doped mesoporous hollow CQD (NCQD) with outstanding thermal conversion efficiency and great fluorescence imaging property has been created and used to monitor PTT’s healing progress [146]. Another variety of CQD is the graphene QD (GQD). For the targeted delivery of Pt to OSCC cells, a polyethylene-glycol-GQDs-Pt (GPt) nanocomposite was found, which showed that GPt might significantly increase the efficacy of chemotherapy for OSCC under both normoxia and hypoxia circumstances [147].

## 12. Nanotechnology-Based Carriers for OC Therapy 

Molecularly targeted medicines are urgently needed to tackle problems with traditional chemotherapeutic agents through increasing medication efficacy and lowering possible toxicity. In nanodelivery systems, drug-loaded NPs of an ideal size can direct the keen modification of drug release behaviours once the microenvironment is changed to some extent, which is used for targeted drug delivery therapy. Drug carriers based on nanotechnology have made it possible to use selected treatment methods for OSCC [1,148]. Targeted drug delivery systems can significantly increase the key characteristics of the bioactive agent (AMDE—absorption, metabolism, distribution, and elimination) with reduced side effects. Nanotechnology has advanced the production of nanocarriers made from organic and inorganic materials, such as liposomes, micelles, hydrogel, and liquid crystals [149]. Through making drugs more effective, they have demonstrated significant promise in the treatment of cancer. These biocompatible/biodegradable polymers include PEG, poly (-benzyl glutamate), polyD, L-lactide, polylactic acid, poly D, Lglycolide, polylactide, co-glycolide, gelatin, sodium alginate, polycyanoacrylate, chitosan, polysaccharides, and proteins. Recent PNPs have also been made from these materials. They demonstrated physical stability, controlled release, and great tolerability, protecting the included labile medicines from degradation. As a result, they can be administered via many different routes, including oral, ophthalmic, cutaneous, pulmonary, and rectal [150].

## 13. DNA-Based Molecular NPs 

For many years, DNA nanostructures have been used to treat a variety of diseases [151]. Tetrahedral DNA nanostructures (TDNs) have demonstrated superiority over other nanostructures among these with diverse forms for a variety of reasons, including acceptable permeability, low cytotoxicity, and relative stability [152]. TDNs are now prospective medication delivery systems as a result of the aforementioned reasons. Despite their relative durability, DNA nanostructures may still be damaged by enzymes and MPS, which limits the applicability of drug delivery carriers in vivo. The cell membrane might also be a challenge for some therapeutic targets that are found inside cells [153,154]. To address these issues, Tian et al. utilised ethylene imine to enhance the characteristics of TND [155]. The traditional gene delivery carrier, ethylene imine, has been widely employed for more than 20 years [156,157]. Researchers created a combination of ethylene imine and TDNs that enhanced lysosome escape and cell entrance while acting as a DNA protector. TDNs are thought of as multifunctional components that have the ability to load different functional units. The novel TDN complex thus holds potential as a candidate for targeted medication delivery in the treatment of cancer. It is well established that TDNs’ cell permeability and drug loading efficiency are important determinants of therapeutic efficacy; as a result, these characteristics must be enhanced. 

Similar to TDNs, researchers have focused extensively on nucleoside NP-based drug delivery systems, particularly in the field of cancer therapy. The creation of a monomeric self-assembled nucleoside NP (SNNP) that was loaded with 5-FU and both in vitro and in vivo tests validated the SNNP’s good biocompatibility and low toxicity [158]. In a subsequent investigation, it was discovered that SNNP improved the stability and concentration of 5-FU in tumour tissues, increasing the tumour-targeting impact. 

In recent years, DNA-based gadgets using bottom-up [159] manufacturing and nanotechnology-mediated DNA material architectures have become intriguing. The semiconducting characteristics of DNA-based devices powered by external electric and magnetic fields gives them potential. Nanomachines, nano-templates, and nano-electronic materials (one-dimensional molecular wires) are only a few examples of applications based on DNA architectures that have been proven [160,161,162,163]. Additionally, because of their exceptional similarity, the electron transmission across such molecular wires and DNA strands may be modelled as donor–bridge–acceptor systems [164]. Previously, DNA-based biosensors were used for temperature sensing [165,166], magnetic particle [167] detection, gene analysis, genetic problem detection, and tissue matching [168,169,170]. Once MDM sandwiches are constructed, these lines enable charge transfer function as rectifiers, transistors, or switches [171,172,173,174,175]. In fact, DNA is a fantastic substitute for silicon when using molecular-scale technology. DNA’s effectiveness in nano-electronic devices, either as a template for manufacturing nanocircuits or as a component of such circuits, is a key factor in the growing interest in this technology. Without a doubt, the development of nanotechnology would be significantly impacted by a feasible conductive DNA variation [164,176,177,178,179,180]. Molecular electronics has made significant advancements in a number of rectifier-related problems. The placement of the chromophore between the two metal electrodes, the work function differences between two different metals or the metal–molecule interfaces, and the molecular orbitals acting as various sources that originate the asymmetric V-I response are a few of these notable issues [181,182].

Through creating a simple electrical device based on a single organic molecule composed of a donor-sigma bond-acceptor, Aviram and Ratner developed the concept of electronic rectification of DNA in 1974 [183]. Later, electrical transmission between two platinum (Pt) electrodes was discovered by Porath et al. [184,185]. Kasumov and colleagues then showed how DNA molecules behave in an Ohmic manner [186]. De Pablo et al. [187] recognised the impacts of dc sequence on DNA conduction characteristics. Additionally, Porath [188] proposed several techniques for detecting lone DNA molecules on a substrate under ambient circumstances. Despite several challenges, there is a lot of research being performed on magnetic biosensors and magneto-resistive bio-chips because of their potential as a particular bio-system capable of attaining high throughput, short delay time, and significant sensitivity [189,190,191,192,193]. In different magnetic field strengths, Ferreira et al. investigated the relationship between the DNA target and the AC field [194]. We recently reported on the variations in the I–V characteristics of the GDG structure with constant G-G gaps in the presence and absence of external magnetic fields with strengths below 1200 mT. But it has never been documented how different gaps affect the I–V properties of GDG structures.

## 14. Cyclodextrins for OC Therapy

The cyclic oligosaccharide family known as cyclodextrins (CDs) are produced when starch is broken down by enzymes. CDs can interact with hydrophobic guest molecules, including docetaxel, cisplatin, methotrexate, and paclitaxel, to form complexes [195]. The external polar surface aids in solubilizing actions in aqueous solutions, whereas the interior lipophilic cavity precludes hydrophobic molecules. Thus, these cyclodextrins and their derivatives are widely used as adaptable, multifunctional excipients for a targeted drug delivery system with high therapeutic efficiency and pharmacological activity [196,197]. Through the use of phospholipid compound technology and a hydroxypropyl-beta-cyclodextrin (HP—CD) inclusion approach, Wang et al. discovered a type of soluble supramolecular complex that ostensibly increased the solubility and oral bioavailability of two curcuminoids [198]. These supramolecular complexes had considerable potential for oral cargo delivery since they were easy to make and had improved gastrointestinal absorption capability.

## 15. Nanolipids for OC Therapy

Although NPs are widely used to treat OC, their potential cytotoxicity and slow uptake by tumour cells reduce their efficacy [199]. To get around this restriction for the treatment of OC, nanolipid-based carriers are easily manufactured and often used. These amorphous clusters of local chemopreventive medicines can be accommodated in these nanostructured lipid carriers’ distorted crystalline structures, which are made of solid and liquid lipids within a core matrix [200]. Nanolipids can enhance the bioavailability, solubility, and stability of drug carriers for therapeutic OSCC applications based on these benefits [201,202,203].

## 16. Peptides/Proteins for OC Therapy

Additionally, synthetic peptides are available for oral targeted delivery. Because it can target goblet cells, the CSKSSDYQC (CSK) peptide is frequently used to enhance hypoglycaemic impact. Investigations have discovered that intestinal goblet cells were the focus of CSK peptide-decorated chitosan NPs, which effectively increased the oral bioavailability of other peptides and small medicines through encouraging intestinal cellular absorption for oral distribution [204]. Functional peptide-conjugated transferrin receptor-specific nanocarriers increased intracellular absorption, changed intracellular trafficking, and enhanced transcytosis in polarised cells for targeted oral drug administration [205].

## 17. Virus-like Particles for OC Therapy

Virus-like particles (VLPs) are often created when viral capsids or envelope proteins produced from viruses self-assemble. Due to their surface biophysical and chemical characteristics, VLPs are easily controlled through genetic and chemical engineering to give them multifunctionality [206]. They have been extensively studied to determine their efficacy as oral antigen carriers for immunisation, but it is still unknown whether they have superior delivery qualities for alternative treatments for OC [207,208,209].

## 18. Future Perspective

The most frequently researched nanotechnologies in cancer diagnosis and therapy are liposomes, dendrimers, magnetic NPs, polymeric NPs, QDs, and gold NPs. Chemotherapy and PDT/PTT are the most frequently used therapeutic modalities. Cancer chemotherapy is developing rapidly, and new medications are being released onto the market more frequently. Unfortunately, OC treatment has not yet yielded good outcomes. Its development has been hampered by inefficiency, a variety of adverse effects, toxicity against healthy cells, and other issues [210,211]. It is encouraging that drug delivery methods based on nanotechnology provide the possibility of improving therapeutic effectiveness for OC, with benefits such as excellent bioavailability, tailored distribution, and minimal side effects. Novel therapeutic agents therefore promise to eliminate as many cancer cells as possible without harming healthy cells. Thus, individuals with OC can have an improved survival rate and quality of life.

The foundation of PDT is the photochemical processes that lead to the production of cytotoxic reactive oxygen species in response to light. There are several issues that need to be resolved, such as phototoxicity, low permeability, and limited efficiency, despite the fact that it is an effective cancer therapy. Similar to PTT, the outcomes are insufficient to permit the extension of its uses [212]. The fact that these challenges have been overcome in some ways thanks to the efforts of researchers is heartening. In a nutshell, improvements in medication safety, treatment effectiveness, and controlled drug release have improved PDT/prospects. Despite PTT’s immense potential in the use of nanotechnology-based drug delivery systems for the treatment of OC, numerous problems still exist, including limited drug encapsulation efficiency, the buildup of nanomaterials in the body, and high preparation costs [213,214,215]. As a result, there is a pressing need to enhance the qualities of nanodrugs and establish official standards for therapeutic use. Governments, health organisations, and researchers should all collaborate on this project.

The development of a drug delivery system based on nanotechnology has drawn more interest and has expanded to include research on numerous kinds of cancer. The liposome is a promising option for usage as a nano-drug, as was described before. In another recent research project, Ruttala et al. produced a multi-polysaccharide-drug complex-loaded liposome NP against the same illness, building on the siRNA-loaded hybrid liposome [216]. The development of numerous other new liposome NPs to improve the treatment of various tumours has yielded hopeful results. Additionally, inorganic NPs are emerging as viable cancer treatment options. Although these modern nanotechnologies show promise in the treatment of tumours, they have just recently been applied to the treatment of OC. Therefore, there is still much effort to be applied to develop nanodrugs that are more potent against OC. In order to speed up the clinical uses of several previously studied nanotechnologies, it is necessary to confirm their availability. Finally, nanotechnology has enabled significant advancements in cancer immunotherapy. Immunotherapy, in contrast to conventional methods, aims to improve the host’s immune capacity so that it can detect, identify, and eliminate cancer cells, which offers new insights into the development of nanomedicine. As was mentioned above, numerous attempts have been undertaken to lessen the uptake of nanodrugs by MPS; however, immune cell-targeting nanomedicines may be able to get around this issue through a different design. Additionally, the immune system that nanomedicines target may continue to have long-lasting impacts on the host to lessen tumour return. Explorations of immuno-nanomedicines for cancer therapy have started to arise in light of these possible benefits.

## 19. Conclusions

Nanotechnology-based medication delivery systems show enormous promise for use in the prevention and treatment of OC. However, because the majority of research is carried out using animal models, little is known about the safety of medication delivery systems based on nanotechnology. The application and promotion of these techniques are somewhat constrained by the technical complexity and high expense of diagnostic and treatment tactics mixed with nanotechnology. Together, researchers and medical professionals must clarify more nanomedicine mechanisms in order to create a standardised approach to using nanotechnology to treat OC. We anticipate seeing its wide-ranging practical uses become available at lower prices, enabling early detection of OC and improved treatment outcomes.

The use of nanoparticles in drug delivery systems is a topic of considerable research interest. The distinguishing characteristics of nanoparticles include their small size, high surface area to mass ratio, and functional structure. These characteristics are investigated for a variety of benefits, including extended circulation duration, medication delivery at the target location, increased bioavailability, and less toxicity. Through examining the constraints on nanoparticle techniques and maximising the existing potential of nanoparticle formulations and technologies, the difficulties in targeted drug delivery systems using nanoparticles can be overcome.

## Data Availability

The data presented in this study are available in this article.
